# Unveiling new chapters in medullary thyroid carcinoma therapy: advances in molecular genetics and targeted treatment strategies

**DOI:** 10.3389/fendo.2024.1484815

**Published:** 2024-10-08

**Authors:** Jia-Xuan Huai, Fang Wang, Wen-Hui Zhang, Yan Lou, Gao-Xiang Wang, Li-Ji Huang, Jing Sun, Xi-Qiao Zhou

**Affiliations:** ^1^ Department of Endocrinology, Jiangsu Province Hospital of Chinese Medicine, Affiliated Hospital of Nanjing University of Chinese Medicine, Nanjing, China; ^2^ The First School of Clinical Medicine, Nanjing University of Chinese Medicine, Nanjing, China; ^3^ Department of Otolaryngology, Xinyang Central Hospital, Xinyang, China

**Keywords:** medullary thyroid carcinoma, molecular genetics, RET gene, RAS genes, targeted therapy, biomarkers, immunotherapy, multi-kinase inhibitors

## Abstract

Medullary Thyroid Carcinoma (MTC), a neuroendocrine malignancy that arises from the calcitonin-secreting parafollicular C-cells of the thyroid, constitutes a minor yet impactful fraction of thyroid malignancies. Distinguished by its propensity for aggressive growth and a pronounced tendency for metastasis, MTC poses formidable obstacles to the early diagnosis and therapeutic intervention. The molecular genetics of MTC, particularly the role of the RET gene and the RAS gene family, have been extensively studied, offering insights into the pathogenesis of the disease and revealing potential therapeutic targets. This comprehensive review synthesizes the latest advancements in the molecular genetics of MTC, the evolution of precision therapies, and the identification of novel biomarkers. We also discuss the implications of these findings for clinical practice and the future direction of MTC research.

## 1 Introduction

Medullary thyroid carcinoma (MTC), a highly invasive neuroendocrine neoplasm, originates from the thyroid’s parafollicular C-cells, which originate from the neural crest and are characterized by their secretion of calcitonin (CTN)-a pivotal biomarker for the identification and surveillance of MTC (1). The disease occurs in both hereditary and sporadic forms. The inherited form, which constitutes 25% of cases, typically exhibits autosomal dominant inheritance and is associated with multiple endocrine neoplasia type 2 (MEN2) syndromes, and the sporadic form makes up the remaining 75% of cases (2). Three distinct variants of the MEN2 spectrum, categorized as MEN2A, MEN2B (alternatively designated as MEN3), and Familial Medullary Thyroid Carcinoma (FMTC), are recognized based on the presence or absence of associated conditions like hyperparathyroidism and pheochromocytoma, along with distinctive clinical manifestations (3). Although MTC represents only 1-2% of all thyroid malignancies, it is linked to considerable morbidity and mortality, characterized by a high incidence of metastasis at the time of diagnosis and a lack of recent advancements in early detection or patient survival rates (4). Extensive research into the molecular underpinnings of MTC has enhanced our comprehension of its etiology and uncovered possible targets for therapeutic intervention.

## 2 Molecular genetics research

### 2.1 RET gene mutations

The RET proto-oncogene, localized on the chromosomal region 10q11.2, translates into a receptor tyrosine kinase crucial for a multitude of intracellular signaling cascades. This kinase plays a pivotal role in the morphogenesis of diverse organs, encompassing the parathyroid glands, the urogenital system, and tissues that originate from the neural crest, such as the cerebral structures, neural ganglia, intestinal ganglia, adrenal medulla, and the calcitonin-producing C cells of the thyroid (5). Given its widespread influence, it is not unexpected that dysregulation of RET is implicated in the tumorigenesis observed in multiple endocrine neoplasia (MEN) syndromes, where these organs are commonly affected (6). Within the RET proto-oncogene, a preponderance of identified mutations are missense in nature, predominantly occurring within exons 10 and 11, which form the extracellular portion of the RET protein, or exons 13 to 16, encompassing the tyrosine kinase domain. Furthermore, mutations are not restricted to these regions; exons 3, 5, and 8 are also implicated in the mutational spectrum (1).

In the realm of MTC, the spectrum of over a hundred gain-of-function mutations within the RET gene has been characterized, with germline mutations present in hereditary cases and somatic mutations in sporadic cases (7).

In the context of sporadic medullary thyroid carcinoma (MTC), somatic mutations in the RET gene have been detected in a substantial proportion, ranging from 43 to 71%. Intriguingly, this prevalence escalates dramatically to 85% in cases characterized by advanced metastasis, with the M918T mutation being particularly prevalent (8, 9), followed by C634 within exon 11 (10). RET-mutant MTC cases harboring RET mutations are observed to display a more virulent phenotype, which encompasses an elevated likelihood of lymph node invasion, propensity for distant metastasis, and a generally unfavorable prognosis when juxtaposed with MTC cases featuring alternative mutations (11, 12). This aberrant RET signaling enhances cell proliferation, a critical mechanism for tumor growth (13–15). For a detailed breakdown of the risk levels, mutations, associated diagnoses, and management strategies, please refer to **Table 1**.

**Table 1:** Summary of RET mutations and associated clinical features in Multiple Endocrine Neoplasia Type 2 (MEN2).

Risk level	RET mutation	RET exon	Possible Diagnoses	incidence of PEHO	incidence of HPTH	CLA	HD	Follow-up	Prophylactic Thyroidectomy Recommendations
Highest Risk (HST)	M918T	16	MEN2B	+++	-	N	Y	Physical exam, neck US, serum Ctn, and serum CEA every 6 mos first year, then annually; begin screening for pheochromocytoma at age 11 yr	Within the first year of life or the first months of life based upon specialist and parental discussions. The ability to identify and preserve or transplant parathyroid glands determines level VI dissection.
High Risk (H)	A883F	15	MEN2A	+++	++	N	N	Physical exam, neck US, serum Ctn, and serum CEA every 6 mos first year, then annually. Begin screening for pheochromocytoma at age 11.	At or before age 5 yr, to be determined on the basis of serum Ctn
	C634W/Y	11		+++	-	Y	N		
Moderate Risk (MOD)	G533C	8	MEN2A	+	-	N	N	Evaluate every 6 months for 1 year. Annual follow-ups thereafter if serum Ctn is normal or undetectable. Begin screening for pheochromocytoma at age 16 yr	When serum Ctn becomes elevated or in childhood to avoid lengthy evaluation period.
	C609F/G/R/S/Y	10		+/++	+	N	Y		
	C611F/G/S/Y/W			+/++	+	N	Y		
	C618F/R/S			+/++	+	N	Y		
	C620F/R/S			+/++	+	N	Y		
	C630R/Y	11		+/++	+	N	N		
	D631Y			+++	-	N	N		
	K666E			+	-	N	N		
	E768D	13		-	-	N	N		
	L790F			+	-	N	N		
	V804L/M	14		+	+	N/Y	N		
	S891A	15		+	+	N	N		
	R912P	16		-	-	N	N		

PHEO, Pheochromocytoma - a tumor of the adrenal gland that overproduces catecholamines.

HPTH, Hyperparathyroidism - a condition where there is excessive production of parathyroid hormone.

CLA, Cutaneous Lichen Amyloidosis - a skin condition characterized by amyloid deposits in the skin.

HD, Hirschsprung’s Disease - a congenital condition affecting the large intestine due to the absence of nerve cells.

N, No

Y, Yes

+++, High incidence

++, Moderate incidence

+, Low incidence

-, No incidence

Varies: Incidence varies based on specific mutation.

Various: Applies to multiple mutations and exons.

In hereditary medullary thyroid carcinoma (HMTC), the most common mutations in MEN2A are found in exons 10 and 11, particularly the codon 634 in exon 11, accounting for 85% of mutations in this exon. In MEN2B, 95% of cases exhibit the M918T mutation located in exon 16. This specific mutation is not only linked to the most severe prognosis but also exhibits an exceptionally high rate of heritability, approaching 95% (16).

### 2.2 RAS gene family

Beyond the already recognized RET proto-oncogene, the frontiers of genomic investigation have disclosed a spectrum of supplementary genetic alterations that are instrumental in the etiological framework of MTC.

The trio of N-RAS, H-RAS, and K-RAS genes, collectively known as the RAS gene family, is recognized as a critical oncogenic driver in sporadic MTC, second only to the RET oncogene. Approximately 70% of tumors with a wild-type RET status exhibit RAS gene mutations, predominantly involving HRAS, followed by less frequent KRAS mutations, and with NRAS mutations being quite uncommon (12).These genes encode for 21 kDa GTPase proteins that are instrumental in regulating signal transduction pathways essential for cell proliferation, metabolism, migration, and other cellular activities (17). Significantly, the RAS proteins fulfill their regulatory roles via pivotal signaling cascades, including the Raf/MEK/ERK and PI3K/Akt pathways, which play essential roles in both normal cellular functions and disease states (18, 19).

RAS gene mutations are prevalent in various types of cancer, making up approximately 30% of all somatic mutations found in comprehensive studies of MTC (20). These mutations are significant because they can lead to the dysregulation of the normal functioning of RAS proteins, potentially resulting in uncontrolled cell growth and tumor development (12).

### 2.3 Other genetic mutations

Advanced sequencing techniques, including whole-exome sequencing and next-generation sequencing (NGS), have revealed that in tumors without mutations in the RET and RAS genes, the prevalence of additional recurrent genetic mutations is significantly scarce (20, 21). Nonetheless, some research has pointed to the involvement of alterations in alternative pathways, along with evidence of epigenetic changes in sporadic MTC.

In a subset of sporadic MTC tumors, Furthermore, activation of the mTOR pathway has been identified in sporadic MTC, with a particular increase noted in cases with lymph node metastases. This activation is also observed in RAS-mutant MTC, suggesting a potential role in the disease’s progression (22, 23). Loss of somatic copy number of the CDKN2C gene has been correlated with detrimental clinical consequences, such as an increased propensity for distant metastasis and a markedly diminished overall survival rate. In the absence of this genetic aberration, the median overall survival extends to 18.3 years, which stands in stark contrast to the abbreviated survival of 4.1 years noted among patients with this mutation (24). Furthermore, heightened expression of hepatocyte growth factor (HGF) and its receptor MET has been identified in MTC, potentially indicating a correlation with the occurrence of multifocal tumorigenesis (25).

### 2.4 Epigenetic and post-transcriptional modifications

Epigenetic and post-transcriptional modifications are increasingly recognized as pivotal forces in the pathophysiology of MTC. In a study encompassing 41 individuals with sporadic MTC, a pronounced elevation in the levels of histone methyltransferases EZH2 and SMYD3 was noted in tumors with a more aggressive phenotype. This upregulation was observed irrespective of the mutational landscape of the RET and RAS genes, indicating that epigenetic mechanisms may significantly contribute to the advancement of MTC through pathways distinct from those involving RET and RAS (26).

An assessment of global DNA methylation among peripheral blood leukocytes in individuals with either sporadic or hereditary MTC disclosed that the sporadic variant is marked by an increased level of methylation. This elevated methylation in sporadic MTC might be attributed to environmental factors rather than germline mutations, which are characteristic of hereditary MTC (27).

While mutations in the TERT promoter region are infrequently identified in MTC, an upsurge in TERT gene copy number and the methylation of its promoter region have been documented. Such promoter methylation correlates with heightened TERT expression and telomerase activation, which in turn are associated with diminished disease-free intervals and overall patient survival rates in MTC (28).

MicroRNAs (miRNAs), a subset of non-coding RNA molecules ranging from 18 to 25 nucleotides in length, are recognized as pivotal regulators of gene expression at the post-transcriptional level (29). These miRNAs have been associated with the intricate control of a diverse array of cellular mechanisms and display unique expression signatures that vary between physiological and pathological conditions, as well as in response to a spectrum of therapeutic interventions (30, 31).

Within the realm of MTC, miRNAs have risen to prominence as influential modulators, with particular miRNAs, including miR-21, miR-375, and miR-183, being correlated with adverse clinical prognoses and the perpetuation of metastatic malignancy. In contrast, miR-224 has demonstrated a positive association with the presence of non-invasive disease states and the achievement of biochemical remission (32, 33).

Divergent miRNA expression patterns have been documented between sporadic and hereditary forms of MTC, with a significant reduction in miR-127 levels observed specifically in sporadic cases harboring somatic RET mutations, in contrast to those with the wild-type RET genotype (34). Furthermore, the overexpression of genes involved in microRNA biogenesis, such as DICER, Additionally, an upregulation of genes pivotal to miRNA biogenesis, including DICER, DGCR8, and XPO5, has been identified in tumors with RET mutations; however, this overexpression pattern does not appear to be linked to the mutational status of RAS genes (35).

Emerging research underscores the profound influence of miRNAs on the cellular phenotypes within Medullary Thyroid Carcinoma (MTC). Notably, the suppression of miR-200b and miR-200c in MTC cell cultures has been correlated with the induction of mesenchymal transition, conferring heightened invasive properties. In parallel, the activation of TGFb-1 and the repression of miR-183 have been associated with diminished cellular viability, coupled with an upregulation of the light chain 3B of microtubule-associated protein 1, a key player in autophagy (36). Collectively, these observations illuminate the intricate dynamics of miRNAs in the etiology and advancement of MTC, indicating their potential to act as either promoters or inhibitors of tumorigenesis.

Moreover, the discovery of circulating miRNAs as potential biomarkers in bodily fluids has introduced the concept of “liquid biopsy” for non-invasive disease monitoring (37, 38). These extracellular miRNAs, released from cells into the bloodstream, offer a promising avenue for early detection, treatment response assessment, and monitoring of disease progression in MTC and potentially other cancers (39).

## 3 Molecular genetics therapies

### 3.1 Immunotherapies

Despite initial studies indicating promising outcomes, the efficacy of immunotherapy in patients with MTC has been observed to be relatively subdued compared to its impact on other types of cancer. Recent findings indicate that the immunological characteristics of the MTC tumor microenvironment could be more subdued, or less reactive, than what was initially thought.

In an expanded patient sample of 200 individuals, a thorough examination of co-inhibitory receptors, including PD-1, TIM-3, CTLA-4, LAG-3, and TIGIT, demonstrated a correlation between elevated structural recurrence rates and the presence of TIM-3 and CTLA-4, in conjunction with the concurrent expression of PD-1 and its ligand, PD-L1 (40). Moreover, research by Pozdeyev and colleagues has illuminated the intricate immunological landscape of Medullary Thyroid Carcinoma (MTC), revealing that organized immune cell infiltration is a common feature, identified in nearly half of the primary MTC tumor cases and, more markedly, in an overwhelming majority—90%—of metastatic sites (41). The presence of CD8+ T lymphocytes and B cells was commonly noted, whereas regulatory T cells (Tregs) were identified in less than 5% of the surrounding non-cancerous cells. Recent studies have uncovered CD276 as a prospective immunotherapeutic target in MTC through immune profiling of these tumors (42). CD276 may enhance the efficacy of other immune checkpoint inhibitors. Consequently, the deployment of combination immunotherapy, involving both anti-CD276 antibodies and anti-PD1/PD-L1 antibodies, holds potential as a novel therapeutic strategy in cancer treatment (43).

The immune microenvironment in MTC significantly influences the efficacy of immunotherapies due to its distinctive characteristics. The tumor’s immune cell infiltration patterns, which vary among individuals, are critical for immunotherapy responses, emphasizing the need for personalized treatment approaches tailored to specific immune contexts. Future research should delve deeper into the immunological features of MTC, particularly the distribution and functionality of tumor-infiltrating immune cells, to enhance the potential of immunotherapeutic interventions and optimize treatment outcomes.

### 3.2 Targeted therapies

Multi-kinase inhibitors (MKIs) are therapeutic agents that possess the capability to inhibit multiple tyrosine kinase receptors (TKRs), which are crucial in cellular mechanisms encompassing growth, differentiation, and angiogenesis. These TKRs are integral components of signaling pathways that regulate cell proliferation, including the PI3K/AKT/mTOR and the MAP kinase/ERK pathways. In the context of oncology, TKR mutations or overexpression are commonly observed in cancer cells, contributing to malignant transformation and tumor progression (44, 45). The process of angiogenesis, characterized by the formation of new blood vessels, is essential for tumorigenesis and metastasis, as it supplies vital nutrients and aids in the dissemination of malignant cells to extranodal regions (46). Central to this process are the Vascular Endothelial Growth Factor (VEGF) and its cognate receptor, VEGF-R, which are frequently observed in elevated levels within neoplastic tissues. This overexpression is particularly pertinent to MTC, a malignancy distinguished by an abundant vascular architecture (47, 48). The heightened levels of VEGF and its receptor, VEGFR, observed in MTC, especially in tumors with RET mutations, reinforce the therapeutic rationale for the use of MKIs, alternatively termed anti-angiogenic medications, in the clinical management of advanced-stage MTC.

Selective RET kinase inhibitors are a novel class of highly selective targeted drugs, which are more efficient and safer in clinical application. Notable among the drugs approved in 2020 are selpercatinib and pralsetinib, which have shown remarkable effectiveness in treating patients with prior exposure to tyrosine kinase inhibitors (TKIs) (49). Due to their high selectivity and lower incidence and severity of adverse reactions, selective RET kinase inhibitors are currently a hot topic in the research of MTC treatment.

The pivotal phase 3 LIBRETTO-531 clinical trial has affirmed the superiority of selpercatinib as a premier therapeutic option for individuals afflicted with RET-mutant MTC, demonstrating superior efficacy and safety over cabozantinib or vandetanib. At a median follow-up duration of 12 months, selpercatinib has been shown to notably prolong both progression-free survival and treatment failure-free survival, achieving an 86.8% rate of progression-free survival and an 86.2% rate of treatment failure-free survival at the one-year mark. Moreover, the overall response rate within the selpercatinib cohort peaked at 69.4%, significantly eclipsing the 38.8% response rate documented in the comparator group. Additionally, selpercatinib was associated with fewer dose reductions and treatment discontinuations due to adverse events, highlighting its favorable safety profile in this patient population (50).

## 4 Conclusion

In conclusion, the molecular basis of MTC has been substantially clarified, with key genetic drivers such as RET and RAS gene mutations being identified. The advent of selective RET kinase inhibitors, particularly selpercatinib, has significantly improved treatment outcomes in terms of progression-free survival and safety. However, challenges including therapeutic resistance necessitate ongoing research into resistance mechanisms and the development of innovative combination therapies to enhance MTC treatment efficacy.

While the complexity of MTC’s molecular landscape suggests the presence of additional targets for therapy, the immune microenvironment’s role in MTC also warrants further investigation to refine immunotherapeutic strategies. Overcoming therapeutic resistance is pivotal for the enduring success of targeted therapies, and innovative clinical trials along with combination therapy approaches will be key in this endeavor.

The translation of these molecular insights into clinical practice is crucial for optimizing patient care. As our comprehension of MTC’s molecular genetics deepens, our therapeutic strategies must evolve in tandem. It is expected that ongoing research will yield increasingly personalized and potent treatments, leading to improved patient outcomes and survival rates.
